# Gene Transfer of Prolyl Hydroxylase Domain 2 Inhibits Hypoxia-inducible Angiogenesis in a Model of Choroidal Neovascularization

**DOI:** 10.1038/srep42546

**Published:** 2017-02-10

**Authors:** Anna Takei, Malena Ekström, Parviz Mammadzada, Monica Aronsson, Ma Yu, Anders Kvanta, Helder André

**Affiliations:** 1 Department of Clinical Neuroscience, Section of Ophthalmology and Vision, St. Erik Eye Hospital, Karolinska Institutet, Stockholm, Sweden

## Abstract

Cellular responses to hypoxia are mediated by the hypoxia-inducible factors (HIF). In normoxia, HIF-α proteins are regulated by a family of dioxygenases, through prolyl and asparagyl hydroxylation, culminating in proteasomal degradation and transcriptional inactivation. In hypoxia, the dioxygenases become inactive and allow formation of HIF transcription factor, responsible for upregulation of hypoxia genes. In ocular neoangiogenic diseases, such as neovascular age-related macular degeneration (nAMD), hypoxia seems pivotal. Here, we investigate the effects of HIF regulatory proteins on the hypoxia pathway in retinal pigment epithelium (RPE) cells, critically involved in nAMD pathogenesis. Our data indicates that, in ARPE-19 cells, prolyl hydroxylase domain (PHD)2 is the most potent negative-regulator of the HIF pathway. The negative effects of PHD2 on the hypoxia pathway were associated with decreased HIF-1α protein levels, and concomitant decrease in angiogenic factors. ARPE-19 cells stably expressing PHD2 impaired angiogenesis *in vitro* by wound healing, tubulogenesis, and sprouting assays, as well as *in vivo* by iris-induced angiogenesis. Gene transfer of PHD2 *in vivo* resulted in mitigation of HIF-mediated angiogenesis in a mouse model of nAMD. These results may have implications for the clinical treatment of nAMD patients, particularly regarding the use of gene therapy to negatively regulate neoangiogenesis.

Hypoxia is a stress situation triggering a multitude of responses that ensure survival of organisms to oxygen deprivation. Adaptation to hypoxia occurs by transcriptional upregulation of multiple genes involved in responses such as angiogenesis (e.g. vascular endothelial growth factor; VEGF), formation of red blood cells (e.g. erythropoietin), anaerobic metabolism (e.g. glycolytic enzymes and glucose transporters), and multiple others^1^,^2^. Gene induction in hypoxia is mediated by hypoxia-inducible factors (HIF), a family of heterodimeric transcription factors composed of an α- and a β-subunit capable of recognizing hypoxia-response elements (HRE) in the regulatory regions of hypoxia-inducible genes^3^,^4^,^5^. In contrast to the constitutive HIF-β, oxygen levels regulate HIF-α activity and protein stability. At normoxia, an asparagine residue within the C-terminal transactivation domain of HIF-α is hydroxylated by the factor inhibiting HIF-1 (FIH-1), impairing the recruitment of the coactivator CBP (cAMP response element binging protein)^6^,^7^. An additional modification by hydroxylation regulates HIF-α protein stability, in this instance by a family of prolyl hydroxylase domain proteins (PHD), that hydroxylate two distinct proline residues within HIF-α^8^,^9^,^10^,^11^,^12^. Hydroxylated prolines are the recognition signature for the E3 ubiquitin-ligase von Hippel-Lindau protein (VHL), leading to proteasome-mediated degradation of HIF-α^13^,^14^,^15^,^16^,^17^,^18^,^19^. HIF dioxygenases (PHDs and FIH-1) require molecular oxygen to hydroxylate HIF-α, and are considered the cellular oxygen sensors. Upon oxygen deprivation, the dioxygenases are rendered inactive allowing formation of the transcriptional active HIF. In certain tissues, as the cornea in the eye, avascularity is maintained under hypoxic conditions, illustrating a supplementary regulatory mechanism of HIF-α proteins. In the hypoxic cornea, the tissue-specific inhibitory PAS protein (IPAS; inhibitory Period-Arnt-Sim domain) binds HIF-α subunits and creates a DNA-abortive complex incapable of activating transcription^20^,^21^.

The light sensing retina in the eye is one of the most metabolically active tissues in the human body^22^. A constant oxygen supply warrants the energy demands of the retina^23^,^24^. Choroidal vasculature nourishes retinal pigment epithelium (RPE) and photoreceptors in the outer retina, while retinal vasculature perfuses the inner retinal layers^25^. Lack of oxygen supply can lead to vision threatening pathologies, as in of age-related macular degeneration (AMD), the leading cause of blindness in elderly patients worldwide. Development of AMD is multifactorial and its neovascular form (nAMD) is characterized by choroidal neovascularization (CNV). Cellular and molecular studies have indicated a role for hypoxia in nAMD^26^, with thickening of Bruch’s membrane and drusen formation. In fact, such hypoxic milieu contributes to the stabilization of HIF, and expression of HIF-1α and HIF-2α has been reported in RPE cells of nAMD patients with colocalized elevated VEGF expression^27^, and in mouse models of CNV, expression of HIF in RPE cells has been associated with elevated levels of VEGF and subsequent induction of the angiogenic response^28^,^29^.

In the present study, we have investigated the role of a series of HIF regulatory proteins (PHD1, PHD2, PHD3, VHL, FIH-1, and IPAS) and their ability to negatively regulate hypoxia-mediated responses in RPE cells. Our results show that PHD proteins are the most effective HIF negative regulators in ARPE-19, a model of human RPE cells. Furthermore, we have demonstrated that PHD2 overexpression alone is the best HIF-regulator to reduce HIF-1α protein expression in RPE cells, and sufficient to ablate hypoxia-inducible upregulation of VEGF, and other angiogenesis-related factors and cytokines. Moreover, the negative regulation of HIF-1α in RPE cells stably expressing PHD2 led to a significant impairment on angiogenic responses by endothelial cells *in vitro*, and by ocular vasculature *in vivo* using a novel iris-induced angiogenesis assay. Furthermore, gene transfer of PHD2 *in vivo* to RPE cells, impaired HIF-mediated angiogenesis in a mouse model of induced CNV. These results indicate that overexpression of PHD2 is sufficient to downregulate the hypoxia response in RPE cells, and subsequent angiogenesis, therefore having implications on the development of an innovative molecular approach for gene therapy strategies for the sustainable treatment of CNV present in patients with nAMD.

## Results

### HIF-1α mediates the endogenous hypoxia response in RPE cells

**Figure 1:**
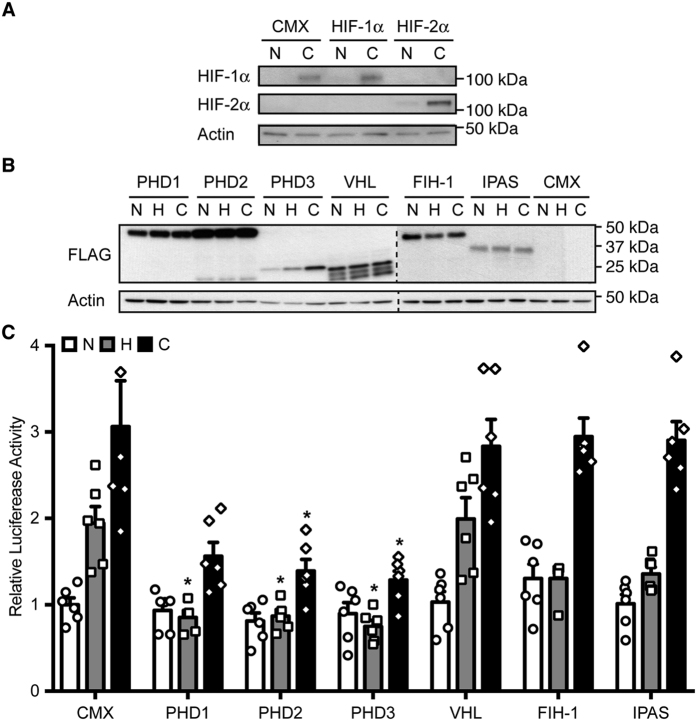
PHDs inhibit hypoxia-mediated transcriptional activation in RPE cells. (**A**) ARPE-19 cells were transfected with pFLAG-HIF-1α or -HIF-2α, or an empty plasmid (CMX), and exposed to normoxia (N) or CoCl_2_ (**C**). Immunoblots demonstrated endogenous levels of HIF-1α, but not HIF-2α, when compared to transfected controls. (**B**) Expression analysis of HIF regulatory proteins in ARPE-19. Cells were transfected with plasmids encoding FLAG-tagged PHD1, PHD2, PHD3, VHL, FIH-1, IPAS, or empty CMX and exposed to normoxia (N), hypoxia (H), or treated with CoCl_2_ (C). (**C**) ARPE-19 cells were transfected with an HRE-driven luciferase reported plasmid and expression vectors for PHD1, PHD2, PHD3, VHL, FIH-1, or IPAS, and exposed to normoxia (N), hypoxia (H), or CoCl_2_ (C). Data are presented as relative luciferase activity to cells transfected with an empty plasmid (CMX) and kept at normoxia. All PHD-expressing plasmids significantly reduced hypoxia-mediated HIF-1α transactivation, and PHD2 and PHD3 also abrogate HIF-1α transactivation in response to CoCl_2_ (mean + SEM, n = 6; *P < 0.05 vs corresponding CMX).

The expression of both HIF-α orthologs in ARPE-19 cells, a commonly used RPE cell line, was investigated. To this end, ARPE-19 cells were transfected with plasmids encoding FLAG-tagged HIF-1α or HIF-2α to generate positive controls for the expression analysis of endogenous HIF-α proteins in this cell line. As presented in Fig. 1A, HIF-1α, but not HIF-2α, could be detected in ARPE-19 cells at the protein level under hypoxia-mimicking conditions (CoCl_2_). These results suggest that, in ARPE-19 cells, the hypoxia-signaling pathway is mediated by HIF-1α.

### Expression of PHDs abrogates hypoxia-inducible transactivation of endogenous HIF-1α in RPE cells

In order to modulate HIF-mediated responses in ARPE-19 cells, expression vectors for FLAG-tagged canonical HIF regulatory proteins (PHD1, PHD2, PHD3, VHL, and FIH-1) and ocular-specific IPAS were assessed. Expression analyses of all FLAG-tagged HIF regulatory proteins displayed considerably high levels of overexpressed proteins upon transient transfection (Fig. 1B).

To evaluate the transactivation potency of endogenous HIF-1α in ARPE-19 HRE-driven reporter gene assays were performed. A hypoxia-inducible transcriptional activation of cells transfected with an empty control was observed in response to either hypoxia or CoCl_2_ treatment (averagely 2- and 3-fold, respectively; Fig. 1C). Generally, HIF regulatory proteins negatively modulate HIF-mediated transcriptional activation in response to hypoxia. Interestingly, upon statistical analysis, all PHD proteins displayed a significant reduction of hypoxia-mediated transactivation (PHD1, P = 0.017; PHD2, P = 0.018; PHD3, P = 0.014), and PHD2 (P = 0.038) and PHD3 (P = 0.029) also significantly reduced HIF-mediated transactivation in the presence of CoCl_2_ (Fig. 1C). These data indicate a demarked role for PHDs in modulating HIF-mediated transactivation in RPE cells.

### PHD2 dramatically decreases HIF-1α lifetime

**Figure 2:**
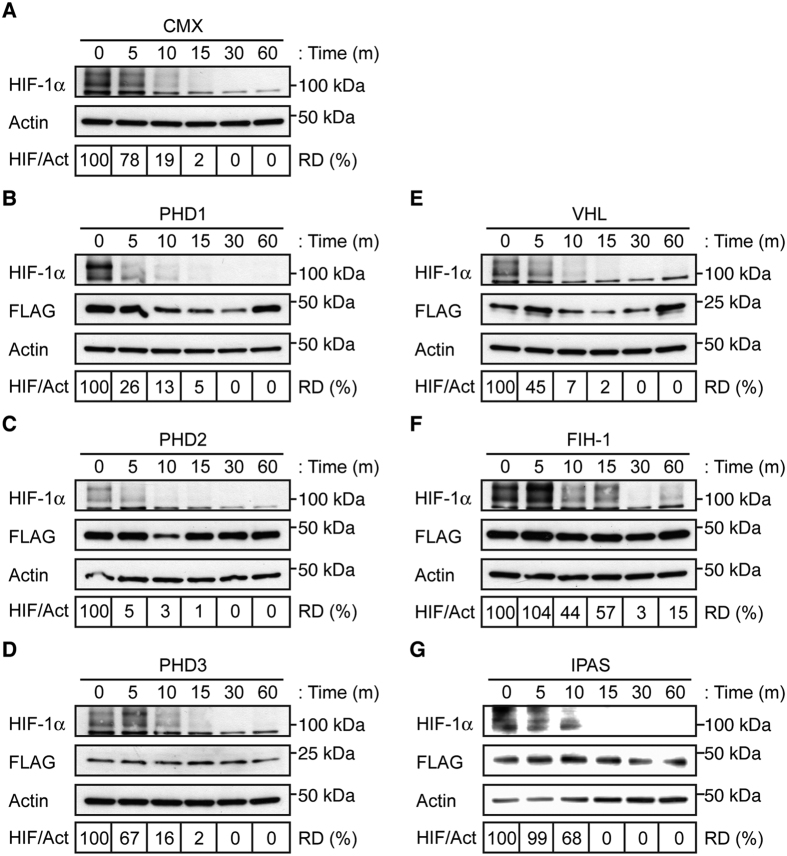
PHD2 overexpression reduces HIF-1α lifetime in RPE cells. Endogenous HIF-1α was stabilized by exposing ARPE-19 cells to hypoxia and the protein lifetime was determined by relative densitometry (RD) of anti-HIF-1α immunoblot in cells transfected with empty CMX plasmid (**A**) under reoxygenation. Analysis of ARPE-19 cells transfected with plasmids encoding FLAG-tagged PHD1 (**B**), PHD3 (**D**), VHL (**E**), FIH-1 (**F**), or IPAS (**G**) did not display reduction of HIF-1α lifetime. Cells overexpressing pFLAG-PHD2 (**C**) showed a considerable reduction on endogenous HIF-1α protein lifetime and expression.

The effect on HIF-mediated transactivation led to investigation of the role of HIF regulatory proteins on endogenous HIF-1α protein in RPE cells. Consequentially, ARPE-19 cells were transfected with each of the HIF regulatory proteins. To allow visualization of HIF-1α protein, ARPE-19 cells were exposed to hypoxia, and the effects of overexpression of HIF regulatory proteins were assayed by determining HIF-1α lifetime on reoxygenation assays, as determined by relative densitometry. Endogenous HIF-1α lifetime was assessed to 10–15 minutes on ARPE-19 cells transfected with empty plasmid (Fig. 2A), as determined by limit of detection on immunoblot. Overexpression of PHDs and VHL reduced HIF-1α protein levels during reoxygenation (Fig. 2A–E), while other HIF regulatory proteins failed to reduce HIF-1α protein levels upon reoxygenation (Fig. 2F,G). In addition, overexpression of PHD2 denoted considerably lower levels of endogenous HIF-1α protein, resulting in a considerable decrease in HIF-1α lifetime. Overall, these data indicate that overexpression of PHD2 alone is sufficient to critically downregulate HIF-1α both at transcriptional activation (Fig. 1C) and by protein levels (Fig. 2C), even under hypoxic conditions, in RPE cells.

### Endogenous levels of HIF-1α protein are reduced in RPE cells overexpressing PHD2

**Figure 3:**
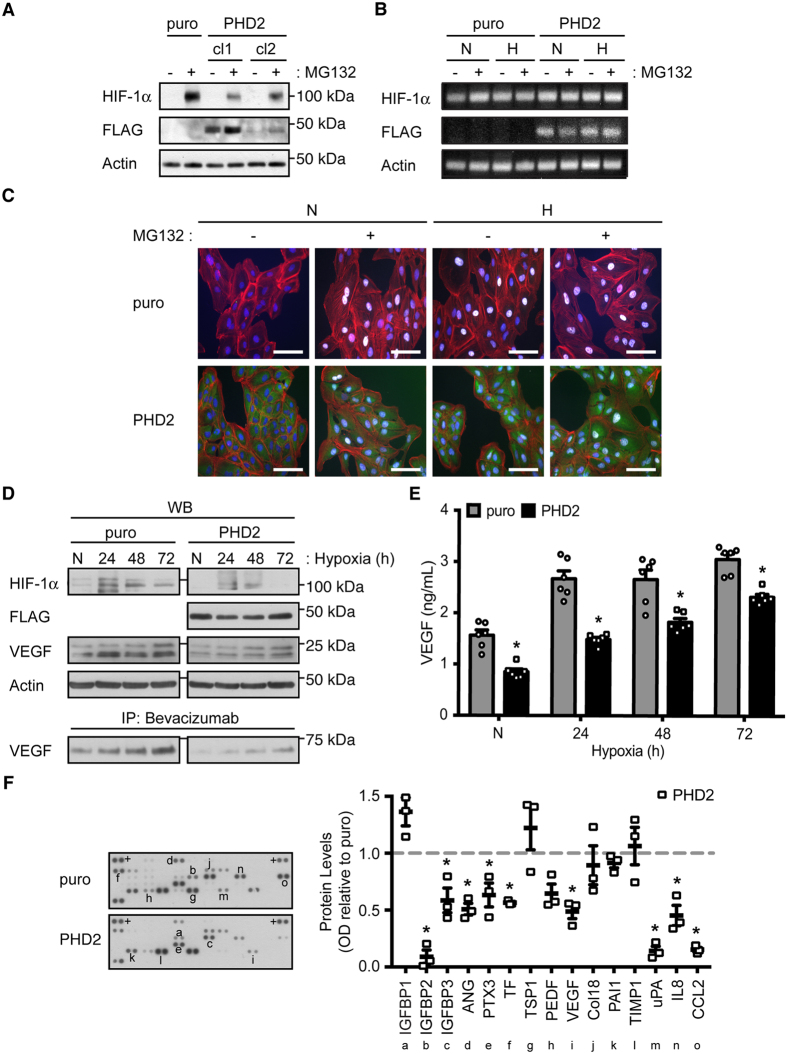
RPE cells stably expressing PHD2 display reduced levels of endogenous HIF-1α and VEGF proteins. (**A**) ARPE-19 cells were transfected and selected to stably express FLAG-tagged PHD2 or an empty puromycin-resistance control (puro). Two clones of RPE-PHD2 cells (cl1 and cl2) were analyzed for HIF-1α and PHD2 protein expression (FLAG), under proteasome blockage with MG132 (+). RPE-PHD2 cells clone 1 displayed high levels of PHD2 expression and considerably lower levels of endogenous HIF-1α protein when compared to the puro control. Clone 1 was used for all subsequent experiments. (**B**) RPE-puro and RPE-PHD2 were exposed to normoxia (N) or hypoxia (H), in the presence (+) or absence (−) of MG123, and HIF-1α transcript expression pattern was analyzed by RT-PCR. No discernable differences could be observed in HIF-1α transcripts in PHD2-overexpressing RPE cells. (**C**) ARPE-19 cells stably expressing FLAG-PHD2 (green) showed reduced immunostaining for endogenous HIF-1α protein (white) in both normoxia (N) and hypoxia (H), even in the presence of MG123, as compared to the RPE-puro control cells. Cells were counter stained to visualize nuclei (blue) and cytoskeleton (red), respectively. Scale bar = 100 μm. (**D**) RPE-puro and RPE-PHD2 cells were exposed to increasing times of hypoxia versus normoxia (N). Immunoblots display a considerable reduction in HIF-1α and intracellular VEGF protein levels in RPE-PHD2 cells. VEGF-capturing assay using Bevacizumab also revealed lower levels of soluble VEGF produced by RPE-PHD2 cells, when compared to the RPE-puro cells. (**E**) VEGF soluble factor was quantified by ELISA in conditioned media from ARPE-19 stably expressing puro control or PHD2 exposed to increasing times of hypoxia. Significantly lower levels of VEGF soluble factor were quantified in media from RPE-PHD2 cells when compared to the RPE-puro control cells (mean + SEM, n = 6; *P < 0.05). (**F**) Angiogenesis-related protein expression profile in media conditioned to 24 h of hypoxia from RPE-puro and RPE-PHD2 cells. Densitometric analysis of predominantly expressed proteins (alphabetized) was preformed against positive controls (+) and presented as expression relative to control (dashed line). A general reduction of angiogenic factors was observed in RPE-PHD2 cells as compared to RPE-puro control cells (mean ± SEM, n = 3; *P < 0.05).

To further study the effects of PHD2 overexpression in RPE cells, ARPE-19 cells stably expressing PHD2 were generated and characterized. After clonal selection by puromycin resistance, two clones of ARPE-19 cells stably expressing FLAG-PHD2 (RPE-PHD2 cells) were compared to one clone of puromycin-expressing control (RPE-puro cells) (Fig. 3A). Clone 1 of the selected RPE-PHD2 cells expressed high levels of FLAG-tagged PHD2 and analysis of these cells by immunocytofluorescence showed a totality of the cells expressing FLAG-PHD2 (Fig. 3C), and was used for all subsequent experiments. Interestingly, RPE-PHD2 cells expressed lower levels of HIF-1α protein, even upon proteasome-mediated degradation inhibition by MG132 (Fig. 3A). We analyzed endogenous HIF-1α transcript expression in RPE-PHD2 and no differences could be observed when compared to RPE-puro (Fig. 3B). When detected by immunocytofluorescence, HIF-1α protein was also reduced in RPE-PHD2 cells both in normoxia and hypoxia, when compared to the RPE-puro control cells, even in the presence of MG132 (Fig. 3C). In sum, the characterization of RPE-PHD2 cells indicates that overexpression of PHD2 in RPE cells induces overall lower levels of endogenous HIF-1α protein.

### PHD2 reduces hypoxia-inducible VEGF expression in RPE cells

To assess the role of PHD2 overexpression on VEGF production and secretion in RPE cells, RPE-PHD2 were compared to RPE-puro cells, upon exposure to prolonged hypoxia (24, 48, and 72 hours). To allow a better comparison between the two stable cell lines, immunoblots with paralleled exposures were presented (Fig. 3D). As expected, the levels of HIF-1α protein were considerably lower in RPE-PHD2 exposed to hypoxia, as compared to RPE-puro cells. Analysis of intracellular VEGF showed an increase in VEGF production in RPE-puro cells exposed to hypoxia but a near steady-state translation of VEGF in hypoxia-exposed RPE-PHD2 cells. To evaluate the levels of secreted VEGF in RPE conditioned media a VEGF-capturing assay was performed, by immunoprecipitating secreted VEGF from the referred media using bevacizumab, a monoclonal neutralizing anti-VEGF antibody. Immunoblot of the precipitated complexes displayed considerably lower levels of soluble VEGF in RPE-PHD2 cell media, as compared to RPE-puro cell media, even in normoxia. Subsequently, soluble VEGF concentrations were quantified in RPE-puro and RPE-PHD2 cells conditioned media by ELISA (Fig. 3E) and confirmed that RPE-PHD2 cells secrete significantly lower levels of soluble VEGF (normoxia, and 24 h and 72 h hypoxia exposure, P < 0.001; 48 h hypoxia exposure, P = 0.002). To evaluate the effect of overexpression of PHD2 in angiogenic factors secreted by RPE cells, protein arrays were performed in media conditioned to 24 h hypoxia of RPE-PHD2 cells and compared to RPE-puro control (Fig. 3F). Analyzed proteins where grouped into insulin-like growth factor (IGF) signaling (IGF-binding proteins; IGFBP), vascular regulation (angiogenin, ANG; pentraxin-related protein, PTX; tissue factor, TF; thrombospondin, TSP; pigment epithelium-derived factor, PEDF; VEGF), extracellular matrix (Collagen, Col; plasminogen activator inhibitor, PAI; tissue inhibitor of metalloproteinases, TIMP; urokinase-type plasminogen activator; uPA), and inflammation cytokines (interleukins, IL; C-C motif chemokine ligand, CCL). A significant reduction was observed for IGFBF2 (P < 0.001), IGFBP3 (P = 0.012), ANG (P = 0.015), PTX3 (P = 0.026), TF (P = 0.004), VEGF (P = 0.021), uPA (P = 0.004), IL8 (P = 0.004), and CCL2 (P < 0.001). Interestingly, amongst analyzed proteins, HIF-mediated genes (IGFBP3, TF, VEGF, uPA, IL8, and CCL2)^1^,^2^ displayed over 45% reduction as compared to the RPE-puro control. These finding suggest that overexpression of PHD2 in RPE cells not only reduces hypoxia-inducible VEGF, as well as other HIF-mediated and angiogenesis-associated factors and cytokines.

### Angiogenesis is impaired in HUVE cells in response to RPE-PHD2 cells

The detection of low levels of both intracellular and secreted VEGF in RPE-PHD2 cells, raised the hypothesis that many other proangiogenic hypoxia-regulated factors could be reduced in these cells. To test this hypothesis, two angiogenesis assays were conducted using HUVE cells in the presence of conditioned media from RPE-puro or RPE-PHD2 cells exposed to 24 hours of hypoxia.

**Figure 4:**
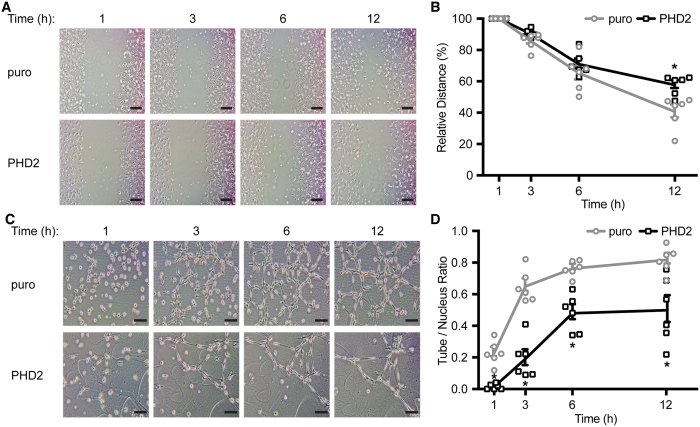
RPE-PHD2 cells impair angiogenesis in HUVE cells. Confluent HUVE cell cultures were scratched to induce wounding or seeded onto matrigel for tubulogenesis, and cultured in media conditioned by RPE-puro or RPE-PHD2 cells exposed to 24 h hypoxia. (**A**) A delay in wound healing is noticeable, particularly at 12 h post-exposure to RPE-PHD2 cells conditioned media. Scale bar = 200 μm. (**B**) Wound marginal distances were measured in HUVE cell cultures. Data is presented as percentage decrease in average marginal distance. RPE-PHD2 cells conditioned medium significantly delays HUVE cells wound healing at 12 h post-exposure, when compared to RPE-puro control cells (mean ± SEM, n = 6; *P < 0.05). (**C**) A considerable reduction in both cell number and tube-like cells was observed in HUVE cells exposed to RPE-PHD2 conditioned medium, when compared to the RPE-puro control cell line. Sale bar = 100 μm. (**D**) Tube-like cells and nuclei were counted in HUVE cell cultures exposed to RPE conditioned media and evaluated through time. Data is displayed as tubes/nuclei ratio. A statistically significant lower tubulogenesis was observed in HUVE cells exposed to RPE-PHD2 cell conditioned media as compared to its correspondent RPE-puro control (mean ± SEM, n = 6; *P < 0.05).

In the wound healing assay, HUVE cell cultures were allowed to recover from a scratch-induced wound in the presence of RPE-PHD2 conditioned medium, and compared to the puro control cell line (Fig. 4A). A general delay in HUVE cells to proliferate and/or migrate into the lesion area was observed in the presence of conditioned medium from RPE-PHD2 cells. When measured, and analyzed as percentage decrease in distance between the two margins of the wound (Fig. 4B), a statistically significant delay in wound healing was observed at 12 hours in HUVE cell cultures exposed to RPE-PHD2 cells conditioned medium when compared to the same time of RPE-puro cells medium-exposed cultures (P = 0.006).

In addition, a tube formation assay was performed using the inherent ability of HUVE cells to form tube-like structures when seeded onto extracellular matrix, such as matrigel. HUVE cells were seeded on to matrigel and exposed to RPE-puro and RPE-PHD2 cells condition media from 24 hours of hypoxia (Fig. 4C). To analyze this experiment quantitatively, the number of tube-like cells was counted, and to avoid any cell number bias, all quantifications were corrected to the total number of nuclei and presented as a ratio of tubes/nuclei. As depicted in Fig. 4D, a significantly lower tube/nucleus ratio was observed in all experimental times (P < 0.001 for all time-points) in HUVE cell cultures in the presence of RPE-PHD2 cell conditioned medium, as compared to the RPE-puro cell conditioned medium.

Taken together, the angiogenesis assays indicate that HUVE cell proliferation, migration, and tube formation are reduced when exposed to medium from RPE cells stably overexpressing PHD2.

### Hypoxia-induced RPE-PHD2 cells mitigate ocular angiogenesis

**Figure 5:**
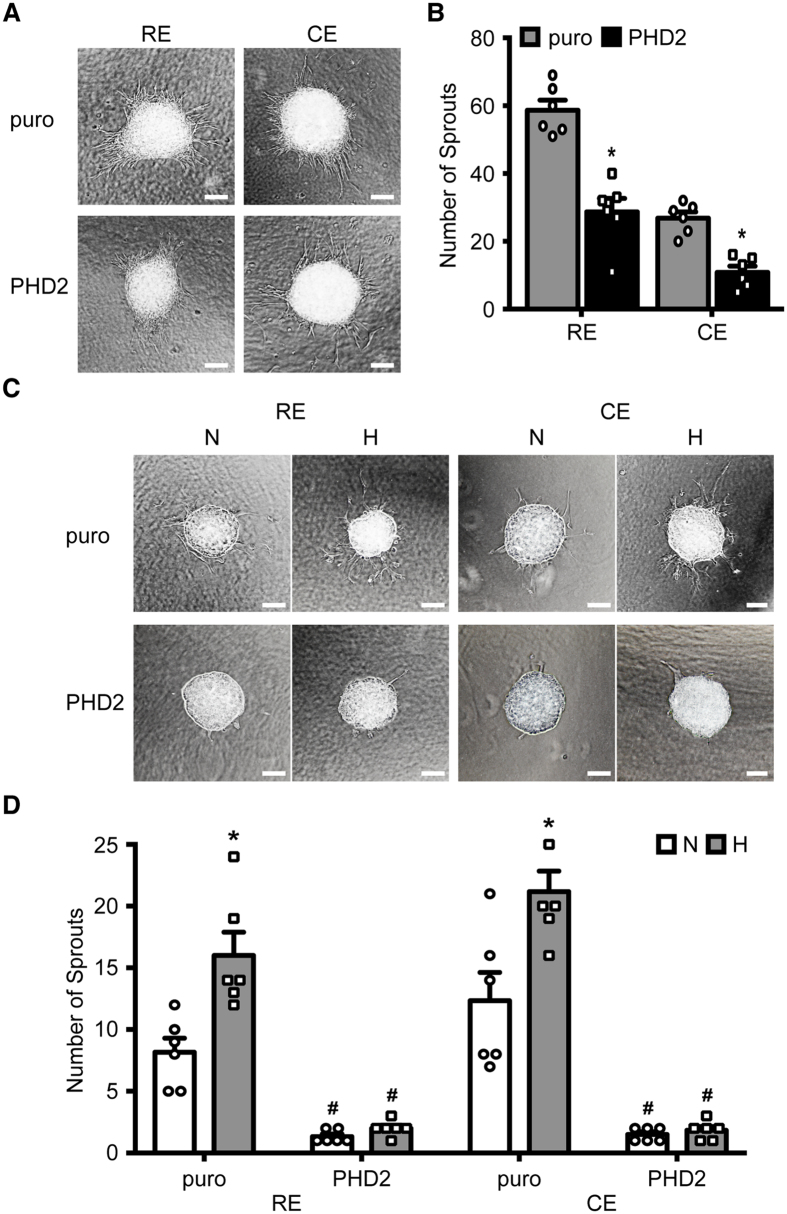
RPE-PHD2 cells reduce angiogenesis in ocular endothelial cells. (**A**) RE or CE spheroids were embedded in matrigel and cultured in media conditioned by RPE-puro or RPE-PHD2 cells exposed to 24 h hypoxia. Scale bar = 100 μm. (**B**) A significant reduction in the number of sprouts were determined in RE and CE spheroids exposed to RPE-PHD2 hypoxia conditioned media when compared to RPE-puro control (mean + SEM, n = 6; *P < 0.05). Data is presented as number of sprouts per spheroid. (**C**) 3D cultures were obtained by mixing cell suspensions of RE or CE cells with either RPE-puro or RPE-PHD2 prior to formation of spheroids, and embedding in matrigel. Cultures where allowed to create sprouts by exposure to normoxia (N) or hypoxia (H) for 36 h. Scale bar = 100 μm. (**D**) 3D cultures containing RPE-puro displayed increased sprouting in hypoxic (H) when compared to normoxic (N) conditions. 3D cultures containing RPE-PHD2 failed to induce sprouts, both in normoxia or hypoxia. A critical reduction of sprouts was observed in 3D cultures containing RPE-PHD2 cells. Data is presented as number of sprouts per culture (mean + SEM, n = 6; *P < 0.05 N vs H; ^#^P < 0.05 PHD2 vs puro).

Likewise experiments with HUVE cells, spheroid cultures of human primary RE and CE cell lines^30^ were established and analyzed for their ability to create angiogenic sprouts when embedded in matrigel. RE and CE spheroids were exposed to RPE-puro or RPE-PHD2 cell conditioned media (Fig. 5A). After 36 h, the number of sprouts was significantly reduced (P < 0.001 for both RE and CE) by approximately 50% in spheroids from either ocular endothelial cell lines exposed to media conditioned by 24 h of hypoxia from RPE-PHD2 cells when compared to RPE-puro-conditioned controls (Fig. 5B).

Being an ocular-specific epithelium, RPE cells are in direct physiognomic contact with retinal and choroidal endothelia. Consequently, to analyze the paracrine responses of RPE cells on ocular endothelia, a new model of 3D cultures was established by creating spheroids where RPE-PHD2 or RPE-puro cells were combined with either RE or CE cells, embedded in matrigel, and kept at normoxia or exposed to hypoxia for 36 h (Fig. 5C). It is important to denote that ARPE-19 spheroids, under similar conditions failed to sprout (data not shown), indicating that the sprouts observed in 3D cultures are endothelial. A near 2-fold increase (RE, P = 0.005; CE, P = 0.011) in number of sprouts was observed in 3D cultures containing RPE-puro cells in both RE and CE cells (Fig. 5D) when exposed to hypoxia. Interestingly, 3D cultures containing RPE-PHD2 cells failed to respond to hypoxia-induced sprouting by the endothelia. Furthermore, endothelial sprouting from RPE-PHD2 cells-containing 3D cultures was significantly reduced (P < 0.001 for all experiments) when compared to puro-containing controls, both in normoxic and hypoxic conditions (Fig. 5D).

**Figure 6:**
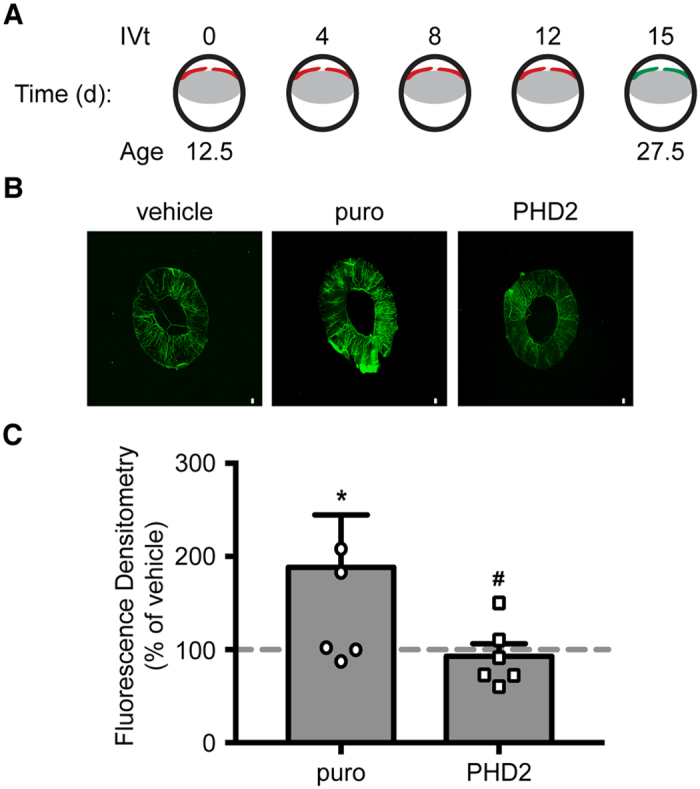
Hypoxic RPE-PHD2 conditioned media mitigates iris angiogenesis. (**A**) Schematic representation of iris angiogenesis protocol. P12.5 mice were IVt injected with RPE-puro or RPE-PHD2 media conditioned by 24 h of hypoxia. Injections were repeated every fourth day posteriorly to the iris (red). At P27.5 mice vasculature was labeled with Dextran-FITC (green). (**B**) Irises were isolated and prepared for whole mount fluorescence. Scale bar = 100 μm. (**C**) Fluorescence densitometry (intensity per area) was determined and presented as percentage of vehicle (dashed line). Media conditioned by hypoxic RPE-puro cells showed an increase in iris angiogenesis, while hypoxic RPE-PHD2 conditioned media presented mitigated angiogenesis (mean + SEM, n = 6; *P < 0.05 vs vehicle; ^#^P < 0.05 vs puro).

To assess the effects of stable expression of PHD2 in reducing secreted angiogenic factors by RPE cells *in vivo*, P12.5 BalbC mice were intravitreally (IVt) injected with RPE-puro or RPE-PHD2 cell conditioned media exposed to 24 h hypoxia, every fourth day for a period of two weeks. The lumen of iris vasculature was labeled with FITC-conjugated high molecular weight dextran by intracardiac injection, and the irises were prepared for fluorescence analysis (Fig. 6A). When compared to vehicle injected mice (Fig. 6B,C), eyes injected with medium conditioned by hypoxic RPE-puro cells displayed a significant increase in iris vasculature (P = 0.039), while eyes injected with RPE-PHD2 cell conditioned medium were statistically undifferentiated from vehicle. Interestingly, vasculature from eyes injected with RPE-PHD2 conditioned medium was significantly reduced when compared to RPE-puro (P = 0.039).

Collectively, these data confirm that RPE cells stably expressing PHD2 produce lower levels of angiogenic factors, even when stimulated by hypoxia, which in turn mitigate angiogenic responses in ocular tissues.

### Gene transfer of PHD2 to RPE cells *in vivo* diminishes ocular angiogenesis

**Figure 7:**
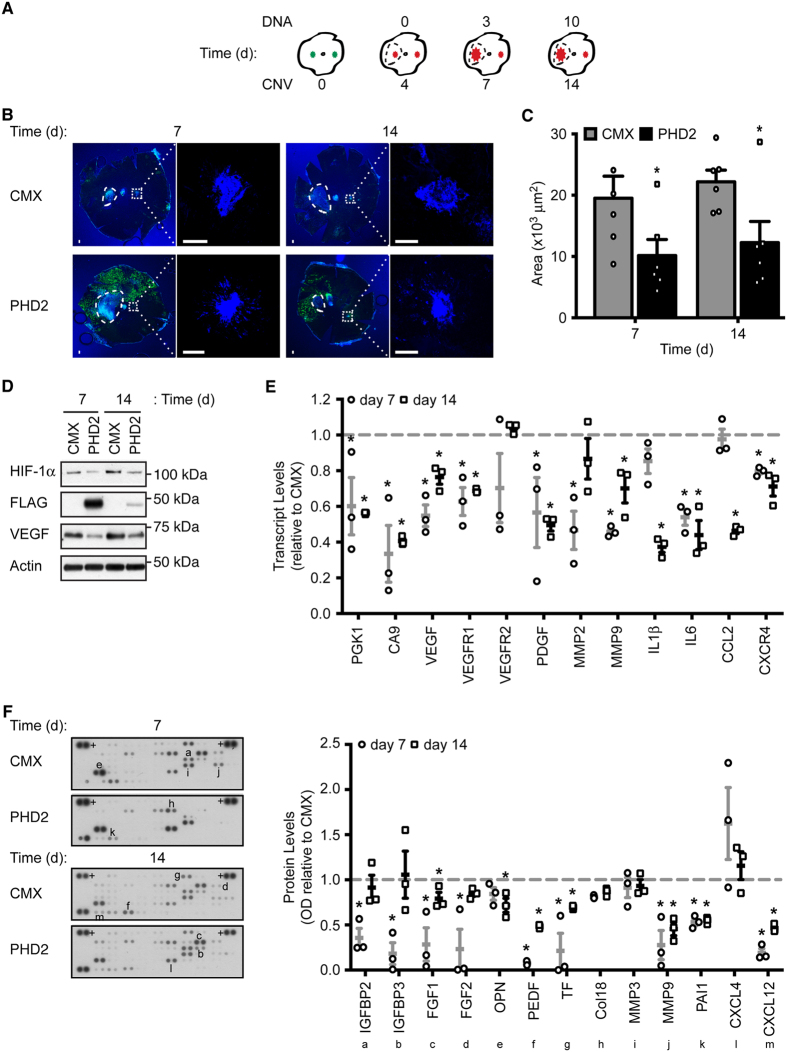
Gene transfer of PHD2 reduces angiogenesis *in vivo*. (**A**) Schematics of laser-induced mouse CNV model with DNA subretinal injection and electroporation. CNV lesions (green) were induced nasally and temporally. Four days post-laser induction the lesions initiate angiogenesis (red) and mice were subretinally injected (dashed circle) with plasmid DNA encoding FLAG-PHD2 or empty CMX. Gene transfer was achieved by electroporation. The creation of a subretinal bleb during DNA injection leads to an increase in the lesion area, while the fellow-lesion (opposing the large DNA bleb) undergoes canonical CNV progression. On days 7 and 14 after laser (equivalent of 3 and 10 days of DNA expression), the posterior eye segments were analyzed. (**B**) Whole-mounts of posterior eye segments were stained with isolectin (blue) and with a FLAG antibody to detect exogenous PHD2 (green). Expression of FLAG-PHD2 expression was observed surrounding the site of injection (dashed circle indicates DNA bleb) on the RPE-side of the posterior eye segments. Fellow-lesion (dotted square) is magnified. Scale bar = 100 μm. (**C**) Quantitative analysis of the fellow-lesion area. A significant reduction of CNV area was observed in fellow-lesions of CNV mice overexpressing PHD as compared to CMX control (mean + SEM, n = 6; *P < 0.05). (**D**) Expression analysis for HIF-1α, VEGF and PHD2 proteins in posterior eye segments of CNV mice expressing control CMX or overexpressing FLAG-PHD2. Increased PHD2 expression correlated with decreased expression of both HIF-1α and VEGF proteins. (**E**) HIF-mediated transcript levels were quantified by qPCR in posterior eye segments of CNV mice overexpressing PHD2. Data is presented as fold (2^−ΔΔCt^) normalized to time-corresponding CMX control (dashed line). Analyzed transcript levels displayed reduced expression in posterior eye segments from PHD2 overexpressing CNV mice (mean ± SEM, n = 3; *P < 0.05). (**F**) Angiogenesis-related factors were profiled in whole-tissue from posterior eye segments from CNV mice expressing CMX or PHD2. Densitometric analysis of proteins of interest (alphabetized) was preformed against positive controls (+) and presented as expression relative to time-corresponding control (dashed line). Angiogenic-factors were reduced in posterior eye segments from CNV mice overexpressing PHD2 (mean ± SEM, n = 3; *P < 0.05).

*In vivo* gene transfer of constructs encoding PHD2 was achieved by electroporation of previously CNV-induced mice (Fig. 7A). Expression of exogenous PHD2 was determined by FLAG-tag (Fig. 7B,D). Albeit transiently, expression of PHD2 was observed during both days 7 and 14 after laser-induction, covering a near 50% of the posterior eye segment area on day 7 and approximately 25–35% on day 14 (Fig. 7B). As assayed by immunoblot, expression of HIF-1α protein was diminished in posterior eye segments overexpressing PHD2 when compared to empty CMX controls, a pattern mimicked by VEGF (Fig. 7D). Moreover, a reduction of CNV area could be observed in eyes expressing exogenous PHD2 (Fig. 7C). Due to the formation of a fairly large subretinal bleb during injection of the DNA, quantitative analysis of CNV lesion area was performed on the fellow lesion, and was significantly reduced (day 7, P = 0.028; day 14, P = 0.031) by a mean decrease of approximately 40%. These data indicate that HIF degradation by exogenous PHD2 is reducing not only VEGF but multiple other soluble angiogenic factors, capable of exerting function even on the fellow CNV lesion.

Consequently, multiple HIF-mediated transcripts were analyzed by qPCR in posterior eye segments of CNV-induced mice overexpressing PHD2 (Fig. 7E). Genes involved in metabolism (phosphoglycerate kinase 1 and carbonic anhydrase 9; PGK1, CA9), angiogenic factors (VEGF, VEGF receptor 1, and platelet-derived growth factor; VEGFR1, PDGF), extracellular matrix degradation (matrix metalloproteinases; MMP2 and -9), and inflammation markers (IL1β and −6, CCL2, and C-X-C motif chemokine receptor; CXCR4) were significantly reduced when compared to CMX controls. Specifically, relative to CMX control transcript levels of most of the analyzed genes were reduced by a mean value of 20–60% on both day 7 (PGK1, P = 0.042; CA9, P = 0.012; VEGF, P = 0.023; VEGFR1, P = 0.007; PDGF, P = 0.048; MMP2, P = 0.010; MMP9, P < 0.001; IL6, P = 0.005; CXCR4, P = 0.047) and day 14 (PGK1, P = 0.020; CA9, P = 0.031; VEGF, P = 0.049; VEGFR1, P = 0.048; PDGF, P = 0.017; MMP9, P = 0.049; IL1β, P = 0.006; IL6, P = 0.008; CCL2, P = 0.046; CXCR4, P = 0.047). Exceptions were denoted for IL1β and CCL2 on day 7, and MMP2 on day 14, when no statistical difference was observed versus CMX. Curiously, VEGFR2 from PHD2-overexpressing posterior eye segments did not statistically differ from CMX controls on either time of analysis.

As previously, angiogenic factors were evaluated by protein arrays using whole-tissue extracts from posterior eye segments of CNV mice overexpressing PHD2 and compared to CMX controls (Fig. 7F). Angiogenic factors analyzed where grouped into cellular growth factors (IGFBP; fibroblast growth factor, FGF), vascular regulation (osteopontin, OPN; PEDF; TF), extracellular matrix (Col; MMP; PAI), and inflammation cytokines (C-X-C motif chemokine ligand, CXCL). A significant reduction at day 7 of CNV was observed for IGFBF2 (P = 0.048), IGFBP3 (P = 0.046), FGF1 (P = 0.015), FGF2 (P = 0.039), PEDF (P < 0.001), TF (P = 0.038), MMP9 (P = 0.044), PAI1 (P = 0.046), and CXCL12 (P = 0.011). At day 14, FGF1 (P = 0.015), OPN (P = 0.017), PEDF (P < 0.001), TF (P = 0.048), MMP9 (P = 0.046), PAI1 (P = 0.035), and CXCL12 (P = 0.050) were significantly reduced. HIF-mediated genes (IGFBP3, FGF2, TF, and MMP9)^1^,^2^ displayed over 50% reduction as compared to the CMX control, particularly on day 7 of CNV when PHD2 expression was highest. Factors such as VEGF and ILs were only detectable at very high exposures (data not shown), and albeit a trend for reduction in PHD2 overexpressing CNV mice was denoted, the data was not considered.

Collectively, these results indicate that during HIF-associated CNV progression, *in vivo* gene transfer of PHD2 expression vectors diminishes angiogenesis by reducing multiple soluble angiogenic-related factors and cytokines.

## Discussion

In the present study, we show the effectiveness of specific HIF regulatory proteins on HIF-mediated transcription activation and protein stability, in RPE cells. Furthermore, we present evidence that, in RPE cells, overexpression of PHD2 alone is the best HIF-regulatory molecule in reducing HIF-1α protein. Consequently, we demonstrate that reduction of HIF-1α protein by PHD2 in RPE cells abolished VEGF hypoxia-inducible expression, promoting subsequent inhibition of RPE-driven angiogenesis, both *in vitro* and *in vivo*, using primary ocular-specific endothelia^30^ and a novel iris-induced angiogenesis assay. Finally, using gene transfer methods, we show that overexpression of PHD2 in an *in vivo* ocular model reduces HIF protein expression and significantly reduces multiple HIF-inducible genes, which culminates in mitigated angiogenesis, and illustrates a novel approach for gene therapy for nAMD.

nAMD is an ocular disease characterized by CNV, where angiogenesis, and particularly hypoxia and VEGF, play an important role^27^,^28^,^29^,^31^. In ARPE-19, we only detect HIF-1α protein, and not HIF-2α, in agreement with previously published work^32^. A third member of the HIF family, HIF-3α, has been described to undergo extensive tissue-specific splicing^33^, and detection of this variant could not be confirmed in ARPE-19 (data not shown). These findings allowed us to focus all subsequent experiments on HIF-1α protein. The HIF pathway has been extensively studied previously^4^,^5^,^6^,^7^,^8^,^9^,^10^,^11^,^12^,^13^,^14^,^15^,^16^,^17^,^18^,^19^,^32^. The present experiments are intended to analyze the effects of overexpression of canonical HIF regulatory proteins (PHD1, PHD2, PHD3, VHL, FIH-1) and the ocular-specific IPAS, with the purpose of identifying the best candidate for anti-HIF gene therapy targeting nAMD. In sum, our studies show that exogenous overexpression of PHD2 alone was sufficient to significantly reduce HIF-1α protein levels in ARPE-19 cells (Fig. 2C and B,D).

A gain-of-function study has previously demonstrated efficacy of PHD2 in reducing hypoxia-inducible endothelial cell proliferation^34^, by interfering with HIF-1α and VEGF protein levels. Analysis of stable overexpression of PHD2 in ARPE-19 cells displayed reduced stability of HIF-1α in the presence of proteasome inhibition, without effects on HIF-1α transcript levels (Fig. 3A,B). These findings could suggest that, rather than an hypoxia-specific regulation, RPE-PHD2 cells maintain a steady-state regulation of HIF-1α by degradation, possibly concomitantly with HIF-1α translation, therefore never allowing proper hypoxia upregulation (Fig. 3C,D). Additionally, lack of hypoxia upregulation of HIF-1α in RPE-PHD2 cells culminates in ablation of hypoxia-inducible intracellular VEGF, and significantly reduces secreted levels of this growth factor to levels compared to normoxia (Fig. 3D,E). Moreover, overexpression of PHD in RPE cells led to reduction on a series of HIF-dependent angiogenic factors and cytokines (Fig. 3F). In fact, HUVE (Fig. 4) and ocular human RE and CE (Fig. 5A,B) cells^30^ exposed to media conditioned by hypoxic RPE-PHD2 cells display impaired angiogenesis. Curiously, albeit some hypoxia-induced *in vitro* angiogenesis could be observed in HUVE, and human RE and CE cells, when exposed to RPE-conditioned media the effects of hypoxia stimulation were statistically insignificant from the experiments conducted in normoxic conditions (data not shown). Such could be explained by the fact that the levels of hypoxia-induced VEGF secreted by ARPE-19 cells greatly exceed the levels secreted by the endothelia cells in this study.

Based on the consideration that IVt injection of large doses of VEGF stimulate iris angiogenesis^35^, we designed a new model of ocular angiogenesis. Pilot experiments in mice pups indicated that wounding of the uveal tissue by sham IVt injections could increase iris vasculature (data not shown), possibly by an indirect increase in angiogenic factors during the wound healing process. Interestingly, when compared to vehicle injected mice pups, the effects of RPE conditioned media are observable in our model of iris angiogenesis (Fig. 6). Albeit VEGF blockage is known to be sufficient to inhibit angiogenesis in endothelial cells^36^,^37^,^38^,^39^, we can postulate that the reduction of secreted VEGF levels in media from hypoxic RPE-PHD2 cells might be mimicked by other HIF-induced proangiogenic factors and contribute for the inhibitory effects observed by us on angiogenesis. In fact, when in the paracrine context of 3D cultures (Fig. 5C,D), RPE-PHD2 cells abolish endothelial sprouting by both RE and CE cells, again suggesting that multiple proangiogenic factors are negatively regulated in RPE overexpressing PHD2.

The intracellular location and molecular function of PHD2 makes it a difficult target for standard pharmacological treatments. In addition, pharmacological substances capable of specifically inducing PHD2 expression or enzymatic activity are lacking. Nevertheless, the development of gene transfer methodologies, particularly in the field of ophthalmology^40^,^41^, made targeting of intracellular molecules and pathways possible. We have previously shown that elevated HIF-1α levels in RPE cells are associated with inducing VEGF expression and lead to induction of angiogenesis in the laser-induced mouse model of CNV^29^. For the first time, we use gene transfer methods by *in vivo* electroporation to overexpress PHD2 in the RPE layer of CNV-induced mice. Analysis of posterior eye segments of CNV lesions in mice overexpressing exogenous PHD2 during the HIF-mediated angiogenic phase display reduced levels of HIF-1α and VEGF proteins (Fig. 7D). Furthermore, analysis of the fellow CNV lesion, away from the increased area of expression of PHD2, display a significant reduction in CNV area, as determined by the endothelial marker isolectin, a sign of reduced angiogenesis (Fig. 7C).

The observation that PHD2 overexpression could inhibit angiogenesis across the posterior eye segments as observed in laser-induced mice suggest that multiple angiogenic factors are suppressed in CNV mice treated with PHD2 gene transfer, as was observed for RPE-PHD2 cells. In fact, HIF-mediated transcript levels associated with CNV in relation to nAMD^25^,^42^ are reduced in posterior eye segments of mice overexpressing PHD2 (Fig. 7E). In agreement with HIF-1α protein expression pattern (Fig. 7D), both PGK1 and HIF-specific CA9 transcript levels are reduced as a consequence of PHD2 overexpression. With regards to VEGF and VEGFR1, the transcript levels reduction displays a slight tendency for higher levels on day 14 after laser-induction when compared to day 7. Albeit reduced versus control CMX gene transfer, a trend of increased expression is also observed for MMPs 2 and 9. Together, and concomitantly with the slight increase in lesion area on day 14 (Fig. 7C), the increase in VEGF family transcripts can be interpreted as a bias of CNV area increase. Curiously, VEGFR2 does not differ statistically from the CMX-expressing control. VEGFR2 increased expression during CNV progression has been particularly associated with CE^29^,^39^, rather than RPE cells. We postulate that in our model, HIF-1α inhibition by gene transfer of PHD2 predominantly targeted the RPE layer while VEGFR2 expression in the posterior eye segments is predominantly choriocapillary. Finally, the general analysis of HIF-mediated inflammation markers associated with angiogenesis and CNV, renders a trend of decreased expression on day 14 after laser-induction when compared to day 7. The role of inflammation in nAMD has been established previously by association of the complement system as a risk factor for AMD^43^,^44^. Furthermore, the mouse genetic model that best relates to AMD initiation and progression was established by CCL2-deficient mice^45^. Our data shows a reduction of inflammation markers in CNV mice overexpressing PHD2, which indicates that regulation of HIF-1α protein levels in CNV can in turn regulate the inflammatory pathways associated with AMD. To further elucidate the possibility that PHD2 overexpression could inhibit overexpression of multiple angiogenic factors and cytokines across the posterior eye segments, protein content was determined by proteome arrays. A general reduction of angiogenic-related proteins could be denoted in the posterior eye segments of CNV mice overexpressing PHD2 (Fig. 7F), particularly on day 7 of CNV when PHD2 peaked in expression with regards to HIF-mediated factors. Together, the observed downregulation of multiple transcripts and proteins involved in angiogenesis-related pathways suggests that negative regulation of elevated levels of HIF-1α protein by gene transfer of PHD2 could modulate pathologic angiogenesis at multiple levels.

Pathologic angiogenesis is associated with a multitude of diseases. In the eye, angiogenesis is involved not only in nAMD but many other sight-threatening conditions, with the common denominator of increased production of VEGF and other angiogenic factors in response to an ischemic insult^23^. The clinical approach of long-standing anti-VEGF treatment of nAMD patients has been associated with increased risk of geographic atrophy^46^, the non-vascular form of AMD. This indicates that inhibition of VEGF alone abolishes CNV but also abnegates the possibility of tissue recovery, resulting in scar formation and tissue atrophy. Our results indicate that negative regulation of HIF-1α in RPE cells by overexpression of PHD2 can simultaneously reduce expression of multiple proangiogenic factors. These findings suggest that gene therapy approaches for regulation of HIF-mediated transcription in RPE cells of patients with nAMD could maintain proangiogenic factors at non-pathologic levels. Such therapeutic approaches could allow for sustainable anti-VEGF treatments, and bypass the risks of multiple injections and development of geographic atrophy. Furthermore, we have shown that transplantation of human embryonic stem-derived RPE cells in a large-eyed animal model of geographic atrophy rescued retinal structure and preserved functionality of photoreceptors^47^,^48^. Combining the results presented here with the results from the transplantation studies, genetic manipulation of human embryonic stem-derived RPE cells to express negative regulatory loops of HIF-1α could present a clinically feasible approach for future treatment of both dry and neovascular forms of AMD.

In conclusion, our results indicate for the first time that overexpression of PHD2 alone in RPE cells is sufficient to reduce HIF-1α and VEGF protein levels, and subsequently reduce RPE-mediated angiogenesis, both *in vitro* and *in vivo*. Consequently, this study indicates that overexpression of PHD2 could be of great value in developing novel gene therapy approaches for sustainable treatment of patients with CNV associated with nAMD.

## Methods

Detailed experimental procedures available as Supplementary Methods.

### Cell culture and transfection experiments

Human retinal pigment epithelium (ARPE-19) and umbilical vein endothelium (HUVE) cells (ATCC, Manassas, VA, USA) were cultured using standard procedures as recommended by the manufacturer. Human retinal and choroidal endothelial (RE; CE) cells were isolated by CD31-positive selection^30^. ARPE-19 cells were transfected with lipofectamine LTX with plus reagent (ThermoFisher Scientific Corp., Waltham, MA, USA) according to the manufacturer’s instructions, using plasmids encoding FLAG-tagged HIF-1α, HIF-2α, PHD1, PHD2, PHD3, VHL, FIH-1, and IPAS, together with the empty plasmid CMX as described in figure legends. Reporter gene assay was achieved by dual-luciferase reporter (Promega, Madison, WI, USA) using a mix of the plasmids pT81–6xHRE-fireflyLuciferase (HRE-Luc) and pCMV-renillaLuciferase (rLuc). Stable transfections of ARPE-19 expressing FLAG-PHD2.puro (RPE-PHD2) or puro control (RPE-puro) were achieved by puromycin (Sigma-Aldrich Corp., St. Louis, MO, USA) clonal selection.

Cells were kept at normoxia in a standard cell culture incubator (20% oxygen, 5% carbon dioxide at 37 °C) or exposed to hypoxia (1% oxygen, 5% carbon dioxide at 37 °C), according to experimental protocols. When described, normoxic cells were treated with cobalt chloride (200 μM CoCl_2_) as a hypoxia-mimicking agent (Sigma-Aldrich Corp.), or with MG132 (10 μM) to block proteasome-mediated degradation (Sigma-Aldrich Corp.).

### RPE medium conditioning and VEGF determination

RPE-puro and RPE-PHD2 cells cultured in reduced medium (1% FBS) were exposed to hypoxia, and media were collected. VEGF-capturing assay was performed using Dynadeads protein G (ThermoFisher Scientific Corp.) immunized with bevacizumab (Avastin; Roche, Welwyn Garden City, UK) onto conditioned media. VEGF quantification was determined by ELISA (Abcam, Cambridge, UK) according to the manufacturer’s instructions.

### *In vitro* angiogenesis assays

HUVE cells exposed to RPE-conditioned media underwent a wound healing assay or a tubulogenesis assay. Wound healing was determined by recovery from a scratch, while tubulogenesis was determined by the number of tube-like cells on a matrigel (BD Biosciences, San Jose, CA, USA) extracellular matrix. Sprouting assay was established on matrigel embedded RE and CE spheroids (alone or in 3D culture with RPE cells). Endothelial sprouts were counted per spheroid.

### Animals

Nine 12.5-day-old BalbC and 36 8-week-old C57Bl6J mice (Charles River, Cologne, Germany) were used in accordance with the ARVO statement for the Use of Animals in Ophthalmologic and Vision Research, and the study protocols were approved by Stockholm’s Committee for Ethical Animal Research. Mice in social groups were housed with a 12 h day/night cycle, free access to food and water, and monitored daily. Mice were euthanized by cervical dislocation, as approved by the ethical committee.

### *In vivo* angiogenesis assays

Iris angiogenesis was assayed in BalbC mice by injecting RPE-conditioned media, and compared to vehicle (non-conditioned medium). Laser-induced CNV in C57Bl6J mice was previously described^29^, and gene transfer to the RPE layer of the retina was achieved by subretinal injection of plasmids and electroporation using a NEPA21 system with a CUY650P5 electrode (NepaGene, Chiba, Japan). After euthanization, irises were analyzed by whole-mount fluorescence, while CNV was analyzed by microdissecting eyes into posterior eye segments, comprehending RPE-choroid-sclera complexes.

### Immunofluorescence and immunoblotting

For immunofluorescence, fixed adherent cells or free-floating posterior eye segments were permeabilized, blocked, and incubated with primary and secondary antibodies per standard procedures. Counterstaining with Hoechst 33258 (Sigma-Aldrich Corp.) and Phalloidin-rhodamine (Biotium), was used when described. Images were acquired using an Axioskop 2 plus fluorescence microscope with the AxioVision software (Zeiss, Gottingen, Germany). For immunoblotting, total protein from whole-cell^49^ or whole-tissue^29^ extracts separated by SDS-PAGE and transferred onto nitrocellulose membranes were analyzed by western blot protocols using enhanced chemiluminescence (ThermoFisher Scientific Corp.). Proteome profiler arrays for angiogenesis (Bio-Techne Corp.) were performed in agreement with the manufacturer’s instructions. When described, ImageJ (NIH freeware) was used to analyze densitometry.

### PCR

Transcript expression pattern from total RNA isolated from ARPE-19 cells (Qiagen, Hilden, Germany) was determined by RT-PCR using Q5 high GC protocols (New Engand Biolabs, Ipswich, MA, USA). Transcript levels from total RNA extracted from posterior eye segments were determined by SYBR green quantitative real-time RT-PCR (qPCR). All qPCR reagents and PrimePCR primer pairs from BioRad Laboratories (Hercules, CA, USA). Data increase or decrease (fold change) of HIF-regulated transcripts was determined relative to CMX control CNV-induced mice of each corresponding day (ΔΔCt method), after normalization to two housekeep genes.

### Statistical analysis

Results are presented as mean ± SEM. Student’s t-test was used for statistical evaluation and P < 0.05 was considered statistically significant. When appropriate, one-way ANOVA was applied for multiple comparisons.

## Additional Information

**How to cite this article**: Takei, A. *et al*. Gene Transfer of Prolyl Hydroxylase Domain 2 Inhibits Hypoxia-inducible Angiogenesis in a Model of Choroidal Neovascularization. *Sci. Rep.* **7**, 42546; doi: 10.1038/srep42546 (2017).

**Publisher's note:** Springer Nature remains neutral with regard to jurisdictional claims in published maps and institutional affiliations.

## Supplementary Material

Supplementary Methods
