# Supplementary material for: Gene Transfer of Prolyl Hydroxylase Domain 2 Inhibits Hypoxia-inducible Angiogenesis in a Model of Choroidal Neovascularization
Source: Sci Rep. 2017 Feb 10;7:42546. doi: 10.1038/srep42546 (PMC5301234; doi:10.1038/srep42546)
Supplement: Supplementary Methods [file srep42546-s1.pdf]

Gene Transfer of Prolyl Hydroxylase Domain 2 Inhibits Hypoxia-inducible Angiogenesis in a Model of Choroidal Neovascularization

Anna Takei, Malena Ekström, Parviz Mammadzada, Monica Aronsson, Ma Yu,
Anders Kvanta, and Helder André

Detailed Experimental Procedures

Plasmid constructs

The plasmids pFLAG-mHIF-1α (HIF-1α), pFLAG-hPHD1 (PHD1), pFLAG-PHD2 (PHD2), pFLAG-PHD3 (PHD3) were described previously[1]. Kind gifts were obtained from Prof. J. Ruas (pFLAG-hFIH-1; FIH-1), Dr. K. Tanimoto (pCMX-hVHL-FLAG), Prof. M. Whitelaw (pT81-6xHRE-fireflyLuciferase; HRE-Luc, and pEF1A-IRES.puro; puro), and Prof. L. Poellinger (pFLAG-mHIF-2α; HIF-2α, and pCMX). pCMV-renillaLuciferase (rLuc) was purchased from Promega. mIPAS (GenBank AF416641.1) was acquired from GenScript (Piscataway, NJ, USA), and subcloned into pFLAG-CMV2 (Sigma-Aldrich Corp., St. Louis, MO, USA) by EcoRI/BamHI. A fragment from pFLAG-hPHD2 digested with EcoRI/(BamHI blunted) was inserted into the pEF1A-FLAG-mHIF-1α.puro[1] linearized by EcoRI/(XbaI blunted), to generate pEF1A-FLAG-hPHD2.puro (PHD2.puro). Frame orientation was confirmed by restriction digestion.

Cell culture

ARPE-19 cells were maintained in DMEM/F12 nutrient mix (1:1) medium containing glutaMAX-1 supplemented with 10 % FBS, and 1 % penicillin/streptomycin mix. Human umbilical vein endothelium (HUVE) cells were grown in F12 nutrient mix containing glutaMAX-1 supplemented with 20 % FBS, 1 % penicillin/streptomycin mix, 0.1 mg/mL heparin, and 0.05 mg/mL endothelial cell growth supplement (ECGS). Reduced medium was composed of F12 nutrient mix containing glutaMAX-1 supplemented with 1 % FBS, 1x Hepes, and 1 % penicillin/streptomycin mix. Both cell lines were purchased from ATCC (Manassas, VA, USA). Human retinal and choroidal endothelial (RE; CE) cells were isolated by CD31-positive selection as previously published[2]. Both RE and CE cultures were maintained in F12 nutrient mix containing glutaMAX-1 supplemented with 10 % FBS, 1 % penicillin/streptomycin mix. All cell culture media and supplements from ThermoFisher Scientific Corp. (Waltham, MA, USA), with the exception of heparin and ECGS (Sigma-Aldrich Corp.).

Transient transfections

ARPE-19 cells were transfected with lipofectamine LTX with plus reagent

(ThermoFisher Scientific Corp.) according to the manufacturer's instructions. Cells were kept at normoxia in a standard cell culture incubator (20 % oxygen, 5 % carbon dioxide at 37° C) or exposed to hypoxia (1 % oxygen, 5 % carbon dioxide at 37° C), according to experimental protocols. When described, normoxic cells were treated with cobalt chloride (200 μM $CoCl_2$) as a hypoxia-mimicking agent (Sigma-Aldrich Corp.), or with MG132 (10 μM) to block proteasome-mediated degradation (Sigma-Aldrich Corp.).

Protein expression analysis

ARPE-19 cells were aliquoted into 6-well plates and subsequently transfected with 500 ng of plasmid DNA (pDNA) encoding FLAG-tagged HIF-1α, HIF-2α, PHD1, PHD2, PHD3, FIH-1, VHL, or IPAS; plasmid concentrations were kept normalized to 1000 ng with empty pCMX vector. Cells were allowed to recover for 24 h and, as depicted in figure legends, kept at normoxia or exposed to hypoxia or treated with $CoCl_2$ for 16 h. Whole-cell extracts and immunoblots are described below.

Reporter gene assay

ARPE-19 cells were pre-aliquoted into 24-well plates and transfected with a pDNA mix containing 280 ng HRE-Luc and 20 ng rLuc, as a reporter system. FLAG-tagged PHD1, PHD2, PHD3, VHL, FIH-1, IPAS, or empty CMX (as a negative control) were added independently at 25 ng of pDNA to the reporter system mix. DNA amounts were equilibrated to a total of 500 ng using pCMX. The cells were allowed to recover for 16 h post-transfection, and exposed to normoxia, hypoxia, or $CoCl_2$ for 24 h. Cells were lysed with 200 μL passive lysis buffer (Promega, Madison, WI, USA), and 40 μL of extract was used for dual-luciferase reporter (DLR) assay (Promega), according to the manufacturer's instructions. Luminometry was carried out using a Tecan Infinity F200 plate reader with dual injectors (Grödig, Austria). Data was normalized to normoxic pCMX DLR ratio.

Reoxygenation assay

ARPE-19 cells were seeded as for protein expression analysis and transfected with 1 µg of PHD1, PHD2, PHD3, VHL, FIH-1, IPAS expression vectors, or empty CMX. All cells recovered for 36 h before exposure to 8 h of hypoxia. After incubation in hypoxia, cells were transferred to normoxia and whole-cell extracts were prepared after 0, 5, 10, 15, 30, and 60 min of reoxygenation. Experiments were analyzed by immunoblotting.

Stable transfections

ARPE-19 cells were transfected in 10-cm diameter dishes with 14 μg pDNA encoding PHD2.puro or empty puro control. Twenty-four h after transfection, cells were exposed to 2 μg/mL puromycin (Sigma-Aldrich Corp.) for clonal selection. Clones of PHD2.puro (RPE-PHD2) or puro (RPE-puro) expressing ARPE-19 cells were analyzed by immunoblotting, immunofluorescence, and PCR in the absence or presence of MG132 and hypoxia for 8 h.

RPE medium conditioning

RPE-puro and RPE-PHD2 cells were grown to 80 % confluence in 10-cm diameter dishes, changed to reduced medium, and exposed to 24, 48, or 72 h of hypoxia, together with normoxia controls. Whole-cell extracts were prepared and the conditioned medium was clarified by centrifugation at 4000 rpm for 4 min at room temperature (RT), aliquoted, zap-frozen in liquid nitrogen, and stored at -80° C until analysis.

VEGF-capturing assay

Twenty μL slurry Dynabeads protein G (ThermoFisher Scientific Corp.) were blocked with 1 % BSA (Sigma-Aldrich Corp.) in Tris-buffered saline (TBS; BioRad Laboratories, Hercules, CA, USA) for 30 min at RT under rotation, followed by immunization with 125 μg Bevacizumab, a clinically used VEGF-neutralizing monoclonal antibody (Avastin; Roche, Welwyn Garden City, UK), for an additional 30 min. Immunization solution was removed and replaced with 1.5 mL RPE-conditioned medium for 1 h under rotation, followed by extensive washes with TBS. Immunocomplexes were eluted from Dynabeads using 20 μL denaturing Laemmli buffer (dLB; BioRad Laboratories) at 95° C for 5 min and analyzed by immunoblotting.

ELISA assay

Fifty μL RPE-conditioned media were incubated overnight (ON) on a VEGF human ELISA kit (Abcam, Cambridge, UK) and analyzed according to the manufacturer's instructions on a Tecan Infinity F200 plate reader equipped with a 450 nm filter. Sample quantification was performed using a regression standard curve, as described by the manufacturer.

Wound healing assay

HUVE cells were grown to confluence on 24-well plates and incubated for 1 h in reduced medium, prior to scratching with a tip to induce wounding. After two phosphate-buffered saline (PBS; ThermoFisher Scientific Corp.) washes, wounded cell cultures were changed into RPE-conditioned media, collected from the 24 h hypoxia-exposed RPE-puro or RPE-PHD2 cells. Images from 1, 3, 6, and 12 h after wounded HUVE cell cultures were exposed to RPE-conditioned media were acquired using a PrimoVert contrast phase microscope (Zeiss, Gottingen, Germany) coupled to a Visicam TC 10 (VWR, Lutterworth, UK). To allow reproducibility, only central field images were acquired and an average of three measurements (top, center, and bottom) of the scratched area using ImageJ freeware were used to assess wound healing.

Tubulogenesis assay

HUVE cells were seeded ($4x10^4$ cells/well) onto 24-well plates pre-coated with growth factor reduced matrigel (BD Biosciences, San Jose, CA, USA), as described by the manufacturer. Cells were allowed to attach for 1 h in reduced medium before exposure to RPE-conditioned media from RPE-puro or RPE-PHD2 cells exposed to 24 h of

hypoxia. To grant reproducibility, central field images were acquired as described for wound healing assay. Tube-like cells and nuclei were counted using ImageJ, and data presented as a tubes/nuclei ratio.

Sprouting assay

RE or CE cells were aliquoted into non-adherent round-bottom 96-well plates ($1x10^3$ cells/well) in medium supplemented with 0.4% methylcellulose (Sigma-Aldrich Corp.) and allowed to form spheroids. Cultures containing spheroids (100 μL) were transferred onto flat-bottom adherent 96-well plates containing 25 μL matrigel, and incubated at 37° C for 1 h. Excess media was carefully removed and replaced with RPE-conditioned media from RPE-puro or RPE-PHD2 cells exposed to 24 h of hypoxia. Alternatively, 3D cultures were obtained by mixing cell suspensions of RE or CE cells with either RPE-puro or RPE-PHD2, at a ratio of 2:1 (endothelium:epithelium) prior to formation of spheroids. After transferred onto matrigel, 3D cultures were incubated in reduced medium. As before, reproducibility was granted by acquiring central field images of 36 h sprouting spheroids and 3D cultures. Sprouts were counted using ImageJ, and data presented as number of sprouts per spheroid.

Animals

Nine 12.5-day-old BalbC and 36 8-week-old C57Bl6J mice (Charles River, Cologne, Germany) were used in accordance with the ARVO statement for the Use of Animals in Ophthalmologic and Vision Research, and the study protocols were approved by Stockholm's Committee for Ethical Animal Research. All mice were acclimatized for approximately 1 week on arrival and housed in social groups of 4-6 animals with litter, sizzle-nest, and housing enrichment. Mice were kept in an IVC system (Allentown Inc., Allentown, NJ, USA) with food and water ad libitum, a light/dark cycle of 12 h, and room conditions averaging 23 °C temperature and 55 % humidity. All animals were monitored daily. At the end of experimental procedures, mice were euthanized by cervical dislocation by trained personal, as approved by the ethical committee.

Iris angiogenesis

BalbC mice were anesthetized using 4 % isoflurane (Baxter, Kista, Sweden) and injected intravitreally with 1 μL reduced medium (vehicle) or 1 μL of conditioned media from RPE-puro or RPE-PHD2 exposed to 24 h of hypoxia. All media was sterilized through a 0.22 μm filter prior to injection. Intravitreal injections were repeated every fourth day. On day 15 after the first injection, mice were deeply anesthetized with ketamine (90 mg/Kg; Pfizer, Cambridge, UK) and xylazine (15 mg/Kg; Bayer, Leverkusen, Germany). One mL of Dextran-FITC (Sigma-Aldrich Corp.; 25 mg/mL in PBS) was injected into the left ventricle of each animal and allowed to circulate for 10 minutes. The animals were euthanized and the eyes were enucleated and cleared from extraneous tissues. After fixing for 6 h at RT in 4 % PBS-buffered formaldehyde (PFA; Solveco, Rosersberg, Sweden), irises were microdissected as whole-mounts for fluorescence microscopy (refer to Immunofluorescence for details). Comparative fluorescence intensity was maintained using fixed camera exposures for all samples. Data were analyzed as fluorescence densitometry (intensity of signal per area) and compared to vehicle.

CNV induction and gene transfer

Two CNV lesions were induced nasally and temporally in C57Bl6J mice as previously described [3]. Four days post-laser induction, mice were anesthetized and subretinally injected with 1 μg of plasmid DNA encoding FLAG-tagged PHD2 or empty CMX in 1 μL endotoxin-free Tris-EDTA buffer (Qiagen, Hilden, Germany). Gene transfer to the RPE layer of the retina was achieved by electroporation using a NEPA21 system with a CUY650P5 electrode (NepaGene, Chiba, Japan). Electroporation was conducted by 3 repeats of a poring pulse (3 pulses of 60 V for 30 ms, with 50 ms interval over 10 % decay rate and dual polarity) followed by a transfer pulse (5 pulses of 20 V for 50 ms, with 50 ms interval over 40 % decay rate and dual polarity). On days 7 and 14 after laser (equivalent of 3 and 10 days of DNA expression), mice were euthanized and eyes microdissected into posterior eye segments, comprehending RPE-choroid-sclera complexes.

Immunofluorescence

For immunocytofluorescence (ICF), ARPE-19 cells stably expressing puromycin resistance or FLAG-tagged PHD2.puro were fixed for 15 min with 4 % PFA at RT. After extensive washes with PBS, fixed cells were permeabilized for 10 min with PBS containing 0.5 % Triton X-100 (Sigma-Aldrich Corp.), followed by 30 min incubation with blocking buffer (10 % normal goat serum from ThermoFisher Scientific Corp., 0.1 % Triton X-100 in PBS) at RT. Primary (1:500 in blocking buffer) and secondary (1:1000 in blocking buffer) antibodies were incubated sequentially for 1 h at RT, with extensive wash steps with PBS following each incubation. Counterstainings (1:5000) for nuclei and cytoskeleton were added to the secondary antibody solution using Hoechst 33258 (Sigma-Aldrich Corp.) and Phalloidin-rhodamine (Biotium), respectively. For immunohistofluorescence (IHF), posterior eye segments followed a protocol similar to ICF with minor modifications: fixation, 30 min; permeabilization, 15 min; antigen retrieval with Diva Decloaker (Biocare Medical, Concord, CA, USA), microwave 3 min; blocking, 1 h; primary antibody step (1:200), ON at 4° C; secondary antibody step (1:500), 1h. Primary antibodies and reagents: mouse anti-HIF-1α (Bio-Techne Corp., Abingdon, UK); rabbit anti-FLAG (Sigma-Aldrich Corp.); isolectin-biotin (ThermoFisher Scientific Corp.). Secondary antibody: goat anti-rabbit-Alexa488; goat anti-mouse-Alexa647; streptavidin-Alexa350 (both ThermoFisher Scientific Corp.). Images were acquired using an Axioskop 2 plus fluorescence microscope with the AxioVision software (Zeiss). For comparison, fixed camera exposures per channel and normalized histogram correction were applied across experiments.

Immunoblotting assays

As previously described, whole-cell extracts were prepared by lysis with RIPA buffer (Sigma-Aldrich Corp.)[1] while whole-tissue extracts were obtained using CelLytic-MT (Sigma-Aldrich Corp.)[3]. Total protein extracts were prepared with dLB, separated by SDS-PAGE, and transferred onto nitrocellulose membranes (all reagents and equipment from BioRad Laboratories). Blots were blocked using 5 % non-fat milk (nfm) in TBS, and incubated with antibodies using 1 % nfm/TBS containing 0.05 % Tween-20 (Sigma-Aldrich Corp.) (TBS-T). For primary antibodies, membranes were incubated with dilutions at 1:500 of: anti-HIF-1α (Bio-Techne Corp.), anti-HIF-2α (Bio-Techne Corp.), anti-FLAG (Sigma-Aldrich Corp.), anti-VEGF (Abcam), anti-Actin (Sigma-

Aldrich Corp.). Secondary antibodies were used at a 1:2000 dilution of anti-rabbit IgG-horseradish peroxidase conjugate (Dako, Carpinteria, CA, USA). After both incubations with antibodies, extensive washes with TBS-T were performed. Visualization of the proteins of interest was achieved using enhanced chemiluminescence (Thermo Scientific) and exposed to hyperfilm ECL autoradiography films (VWR). Due to sample limitations, membranes were cropped into regions of interest, prior to western blot analyses, and data is presented as a full representation of the cropped and blotted area. Paralleled exposures across each figure panel are presented for best comparison. When described, ImageJ (NIH freeware) was used to analyze densitometry.

Protein arrays

Protein profiler arrays (Bio-Techne Corp.) for angiogenic factors and cytokines were preformed according to the manufacturer's instructions. For human soluble factors, 1 mL of 24 h hypoxia-exposed RPE-conditioned media was used. One-hundred μL of whole-tissue extract was used for mouse experiments. Densitometric analysis was determined using ImageJ against two internal positive controls, of the same exposure, across three independent experiments or biological replicates, respectively.

PCR

Total RNA was extracted from ARPE-19 cells or posterior eye segments using RNeasy mini kit (Qiagen), according to the manufacturer's instructions. Generation of cDNA was achieved from 1 μg of RNA by iScript reverse transcriptase (BioRad Laboratories). Cellular transcript expression pattern was determined by RT-PCR using Q5 with high GC enhancer as indicated by the manufacturer (New England Biolabs, Ipswich, MA, USA) with the primer pairs: HIF-1α caccacaggacagtacaggat / cgtgctgaataataccactcaca (25 cycles); FLAG-hPHD2 ggactacaaagacgatgacg / ggttctccatcttcccgcac (35 cycles); Actin gacaggatgcagaaggagat / ttgctgatccacatctgctg (15 cycles). For posterior eye segments, transcript levels were determined by quantitative real-time RT-PCR (qPCR) using iQ SYBR green super mix with gene-specific PrimePCR primer-pairs, on a MyiQ qPCR system (all qPCR reagents and equipment from BioRad Laboratories). Cycle threshold (Ct) of HIF target genes were normalized to the average Ct of 2 housekeeping genes. HIF-regulated transcripts: phosphoglycerate kinase (PGK)1; Carbonic anhydrase (CA)9; VEGF; VEGF receptors (VEGFR)1 and 2; Platelet-derived growth factor (PDGF); matrix metalloproteinases (MMP)2 and 9; interleukins (IL)1β and 6; C-C motif chemokine ligand (CCL)2; and C-X-C motif chemokine receptor (CXCR)4. Housekeep genes: hypoxanthine phosphoribosyltransferase (HGPRT); and TATA-box binging protein (TBP). Data increase or decrease (fold change) was determined relative to empty CMX plasmid electroporated CNV-induced mice of each corresponding day (ΔΔCt method).

Statistical analysis

Immunoblots display representative results of at least three independent experiments from the same exposure. Quantitative in vitro experiments were performed three times with technical duplicates (n=6). Iris angiogenesis was determined in 3 mice per group, and data was quantified from all eyes (n=6). CNV area analysis was quantified from 3 mice as for iris angiogenesis (n=6). Total RNA (and total protein) from CNV mice was

collected from 3 mice as a pool of 2 posterior eye segments from 2 independent animals (n=3). Results are presented as mean ± standard error. Student's t-test was used for paired statistical evaluation while one-way ANOVA with Bonferroni corrected post-hoc tests was used for multiple comparisons, and $P<0.05$ was considered statistically significant.

References

1. André, H. & Pereira, T. S. Identification of an alternative mechanism of degradation of the hypoxia-inducible factor-1alpha. *J. Biol. Chem.* **283,** 29375–29384 (2008).
2. Mammadzada, P., Gudmundsson, J., Kvanta, A. & André, H. Differential hypoxic response of human choroidal and retinal endothelial cells proposes tissue heterogeneity of ocular angiogenesis. *Acta Ophthalmol* **94,** 805–814 (2016).
3. André, H., Tunik, S., Aronsson, M. & Kvanta, A. Hypoxia-Inducible Factor-1α Is Associated With Sprouting Angiogenesis in the Murine Laser-Induced Choroidal Neovascularization Model. *Invest. Ophthalmol. Vis. Sci.* **56,** 6591–6604 (2015).
